# Characteristics of Bread Made of Various Substitution Ratios of Bran Pulverized by Hammer Mill or Jet Mill

**DOI:** 10.3390/foods9010048

**Published:** 2020-01-04

**Authors:** Dabeen Lee, Mi Jeong Kim, Han Sub Kwak, Sang Sook Kim

**Affiliations:** 1 Research Group of Food Processing, Korea Food Research Institute, Jeollabuk-do 55465, Korea; Lee.Da-been@kfri.re.kr (D.L.); hskwak@kfri.re.kr (H.S.K.); 2 Department of Food and Nutrition, Changwon National University, Changwon-si 51140, Korea; mjkim@changwon.ac.kr

**Keywords:** bran milling, hammer mill, jet mill, bran substitution ratio, bread quality

## Abstract

The physicochemical and antioxidant properties of dough and bread were measured in wheat flours substituted with two types of bran (HMB: bran pulverized by a hammer mill and JMB: bran pulverized by a jet mill) at various ratios (0%, 5%, 10%, 15%, 20%, and 25%) of substitution. The particle size of hammer mill bran (HMB) (119.71 µm) was larger than that of jet mill bran (JMB) (25.78 µm). Wheat flours substituted with HMB contained more total dietary fiber than those with JMB. A significant increase of water absorption and dough development time in Mixolab^®^ analysis was observed depending on the level of HMB or JMB substitution. The breads made with HMB or JMB (5% or 10%) showed a higher specific volume and lower crumb hardness than the control bread. However, breads made with ≥15% HMB or JMB had a decreased specific volume and increased crumb hardness. Overall, breads made with wheat flour substituted with 5%–10% HMB or JMB were of a higher bread quality and had more antioxidant properties.

## 1. Introduction

The most important cereal crop suitable for bread is wheat [1]. Wheat consists of three main parts: germs, bran, and endosperm [2]. Wheat flour has a different nutrient composition depending on the extraction rate during milling. Refined wheat flour, produced when the extraction rate is below 75%, is the best type for baking, but it has fewer health benefits than whole wheat flour [1].

Wheat bran is usually eliminated as a byproduct during milling for refined wheat flour, and is used as animal feed. However, the demand for bran is increasing in modern society due to interest in its health benefits: it is richer in nutrients, such as proteins, fat, vitamins, and minerals, than ordinary refined wheat flour, and it contains a variety of physiologically active components [3,4,5].

Bran also contains a large amount of dietary fiber. Proper intake of dietary fiber reduces the risk of obesity, cardiovascular disease, and type 2 diabetes as well as reduces intestinal passage time [6,7]. Therefore, numerous attempts have been made to incorporate wheat bran into various foods, such as pasta [8,9,10], noodles [11], doughnuts [12], and biscuits [13].

In many countries, bread is a staple food, so much effort has been made to optimize the quality of bran-rich bread [14]. However, information on the effects of milling methods and substitution ratios of bran on bakery products is limited. Therefore, the aim of this study was to investigate the effects of milling methods on the characteristics of bran and the effects of the substitution ratio of bran on the properties of bread.

## 2. Materials and Methods

### 2.1. Materials

Bran and flour were provided by CJ Cheiljedang (Seoul, Korea). All reagents used in the analysis were purchased from Sigma-Aldrich Inc. (St. Louis, MO, USA). The wheat bran with 16.64% moisture content was dried to a moisture content of about 6% at 40 °C using a dry oven (HK-DO1000F, Hankuk S&I Co., Hwaseong, Korea) and then milled using a hammer mill (Daega Powder System Co., Ltd., Seoul, Korea) or air jet mill (Daega Powder System Co., Ltd., Seoul, Korea). The milling conditions were as follows: Bran was pulverized by the hammer mill mounted with a 300 μm sifter at a speed of 40 m/s. The air jet mill was run under air pressure of 8 bar with a feed rate of 0.71 kg/h and a vibration rate of the feeder of 70%. The wheat bran pulverized with the hammer mill or air jet mill replaced 0%, 5%, 10%, 15%, 20%, and 25% of the wheat flour weight.

### 2.2. Chemical Analysis

The contents of moisture ash and protein were analyzed by American Association of Cereal Chemists (AACC) Method 44-15A, 08-01, 46-12 (AACC, 2010) respectively, the wet gluten content was determined by the Glutomatic^®^ system (Glutomatic 2200, Perten Instruments, Hagersten, Sweden) as in AACC method 38-12A, and the dietary fiber content was analyzed according to AACC Method 32-07. Starch damage was measured according to AACC Method 76-31 (AACC, 2010) using the Damaged Starch Analyzer (SDmatic, Chopin Technologies, Villeneuve La Garenne, France). The L (lightness) value, the a (redness) value, and the b (yellowness) value were measured using the spectrophotometer CM-700d (Konica Minolta Sensing Americas, Inc, Ramsey, NJ, USA). Particle size was measured by the Particle Size Analyzer 1190 (CILAS, Orléans, France).

### 2.3. Microstructure of Bran and Dough with Various Substitution Ratios of Bran by Scanning Electron Micrograph (SEM)

The microstructure of bran and dough with various ratios of bran was investigated by SEM (S-2380, Hitachi, Tokyo, Japan). The bran and dough were dehydrated as in Inoue and Osatake [15]. The bran or slice of dehydrated dough was placed on an adhesive tape attached to a circular aluminum specimen stub and coated with gold-palladium using Hitachi E-1010 10 N sputter coater (Tokyo, Japan) and photographed at an accelerator potential of 15 kV using SEM.

### 2.4. Evaluation of Dough Characteristics

Mixolab^®^ is a device that measures the characteristics of wheat flour by measuring the torque of the dough as the temperature rises [16]. As dough characteristics, water absorption, lowest viscosity (C2), and maximum viscosity corresponding to starch gelatinization (C3), stability (C4), development time, and retrogradation (C5) of wheat flour were measured using the Mixolab (Chopin, Tripetteet Renaud, Paris, France). The flour was placed in the Mixolab^®^ bowl and water was added to reach a dough consistency (C1) value of 1.1 Nm. Dough development time, indicating the hydration rate of the dough, is the time required for each sample to reach C1, which is an objective value of 1.1 Nm on average. The stability time of the dough indicates the time to maintain a 1.1 torque value. The conditions according to ‘Chopin +’ protocol are as follows: keep the dough at 30 °C for 8 min while mixing, then heat to 90 °C at the rate of 4 °C/min for 15 min. After reaching 90 °C, the temperature was kept constant for 7 min, and cooled to 50 °C at the rate of 4 °C/min for 5 min.

### 2.5. Preparation of Bread and Physical Characteristics of Bread

The bread was made by the modified AACC Method 10-10B (AACC, 2010): 100% of flour, optimum water, 5.3% of yeast, 6% of sucrose, 1.5% of salt, 3% of shortening, and 0.2% of baking improver (Excel, Sunjin, Chungnam, Korea) were added instead of malt flour, ascorbic acid, and potassium bromate. The amount of water added for bread dough was determined based on the water absorption value by Mixolab^®^. The volume, weight, and specific volume of the bread were measured using the Volscan profiler (Stable Micro System Ltd., Haslemere, UK). The hardness of the bread crumb was measured by the modified AACC Method 74-09 (AACC, 2010) with the Texture Analyzer TA-XT plus (Stable Micro System Ltd., Haslemere, UK). The crumb was cut to width × length × height of 25 × 25 × 19 mm, respectively and compressed by approximately 40% using a 36 mm plunger. The return distance was 30 mm, the return speed was 10 mm/s, and the contact force was 50 g.

### 2.6. Free Phenolic Compounds Extraction

The free phenolic compounds of bread were extracted based on the method of Lim et al. [17] with some modifications. The freeze-dried bread (1 g) was extracted with 80% chilled ethanol (20 mL), and the mixture was centrifuged at 8000 rpm for 20 min. After centrifugation, the supernatant was collected, and the residue was extracted with the above procedure twice. The ethanol extract was concentrated using a vacuum evaporator (HS-2001N, Hahnshin S&T Co., Gimpo, Korea), and then dissolved in 10 mL of 80% ethanol. The ethanol extract was stored at −20 °C for analysis.

### 2.7. 2,2′-Azino-bis (3-ethylbenz-thiazoline-6-sulfonic acid (ABTS) Radical Scavenging Activity

ABTS radical scavenging activity was determined by a modified version of the method of Rosa et al. [18]. The samples (40 µL) were mixed with ABTS radical solution (1960 µL), they reacted for 6 min, and absorbance was measured at 734 nm. Each sample activity was expressed in μmol Trolox equivalents (TE)/g.

### 2.8. Oxygen Radical Absorbance Capacity (ORAC) Assay

ORAC assay was determined based on the method of Moore et al. [19]. The samples (20 µL), fluorescein solution in 75 mM phosphate buffer (pH 7.4) (200 µL) and 79.6 µM AAPH (2,2′-azobis(2-amidino-propane) dihydrochloride) (20 µL) were dispensed into a 96-well microplate. After mixing, the fluorescein intensity was measured at excitation of 485 nm and emission of 520 nm (at 1-min intervals) using a Spectra Max^®^ i3 (Molecular Devices, San Jose, CA, USA) equipped with an incubator at 37 °C for 90 min. The result was calculated as the difference between the fluorescein intensity of the sample and the fluorescence intensity of the blank. Trolox was used as the standard material.

### 2.9. Statistical Analysis

Mixolab^®^ assay was conducted in duplicate, the others were conducted in triplicate, and the results were expressed as mean ± standard deviation. Statistical analysis was performed using SPSS 20 (Chicago, IL, USA) software. The effects of the milling methods on the particle size of the bran were analyzed by Student’s *t*-test. Analysis of variance (ANOVA) was carried out to determine the differences in the characteristics of the flour, dough, and bread samples with the different substitution ratios of bran. Duncan’s multiple comparison test was performed to determine the significant differences between treated means (*p* ≤ 0.05).

## 3. Results and Discussion

### 3.1. Composition of Wheat Bran Pulverized by a Hammer Mill or Jet Mill and that of Wheat Flour Substituted with Various Ratios of Bran

The particle size and color of bran pulverized by a hammer or jet mill are shown in Table 1. The particle size of jet mill bran (JMB) was significantly smaller than that of hammer mill bran (HMB) (*p* ≤ 0.05). The air jet mill is a relatively recent technology for milling, and the hammer mill is a traditional impact mill. Pulverization by the air jet mill is made by injecting compressed high-pressure air into a grinding chamber through a nozzle to generate a high-speed spiral air flow and then feeding the raw material into a chamber by a jet nozzle. After that, the suspended particles collide with each other and the particles are released when the particles are milled to the desired size [20]. The hammer mill pulverizes the cereals or objects with the simultaneous exertion of impact force, frictional force, compressive force, and shear force between the hammer and the liner rotating at high speed. The air jet mill achieves an accurate and narrow range of particle size. The hammer mill can control particle size and grind large quantities continuously [20].

The results of the color value (L, a, and b) analysis for bran showed that the L value of the JMB was higher than that of HMB (Table 1). The L, a, and b values were measured as a unit of color, which is represented by one value for brightness and two for color. The color of bran affects the final products with bran. The higher the L value the whiter the color. Therefore, bran pulverized by the jet mill was whiter than that pulverized by the hammer mill. As the particle size gets smaller, the lightness increases because the surface area that can reflect light increases [21]. However, the a and b values of JMB were significantly lower than those of HMB (Table 1). As the ‘a’ value of the sample increases from a negative value to a positive value, the color changes from green to red; as the ‘b’ value of a sample increases, the color changes from blue to yellow. This means that the higher the value of a and b, the more red and yellow the sample is [22].

**Figure 1:**
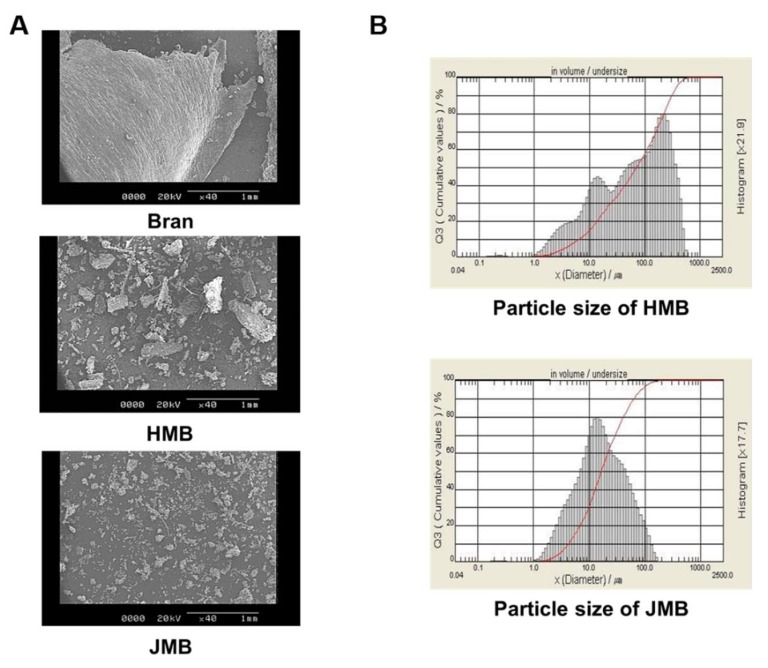
(**A**) Scanning electron microscopy (SEM, ×40) images and (**B**) particle size distribution of HMB or JMB. HMB: bran pulverized by hammer mill, JMB: bran pulverized by jet mill.

The micro images and particle size distribution of bran are shown in Figure 1. Images obtained with scanning electron microscopy (SEM) showed that JMB was uniform in particle shape while HMB was uneven in particle shape, and the distribution of particle size of JMB was narrower than that of HMB, as previously reported by Saravacos and Kostaropoulos [20].

Protein, wet gluten, ash, and total dietary fiber were significantly different depending on the bran substitution ratio (Table 2). As shown in Table 2, the protein content and wet gluten content of refined wheat flour (RWF) were 10.75% and 24.70%, respectively. The ash and total dietary fiber content of RWF were 0.67% and 1.80%, respectively. Bran is one of the outer parts of wheat, rich in dietary fiber and bioactive components [23]. The content of protein, ash, and dietary fiber increased with the substitution ratio of bran. Overall, the wet gluten content decreased with the substitution ratio of bran, while the protein, ash, and dietary fiber content increased with the substitution ratio of JMB or HMB. This is because protein, ash, and dietary fiber content were higher in the bran than RWF [24]. The L value tended to decrease with the substitution ratio of JMB or HMB, while a and b values tended to increase. The b value for the 5% HMB substituted flour tended to increase, like other samples, but did not increase significantly (Table 2). Overall, the results of this study confirmed the report by Chen et al. [25], which showed increased a and b values by the addition of bran.

### 3.2. Dough Characteristics by Mixolab^®^

Dough characteristics measured by Mixolab^®^ are outlined in Table 3. The water absorption of dough increased with the substitution ratio of HMB or JMB. The water absorption of wheat flour is affected by several factors. The water absorption of dough was increased by the addition of bran due to the water binding ability of bran. Since wheat bran competes with other major wheat flour components, such as starch and gluten, for water [26], a higher substitution ratio of bran might result in more water uptake, as shown in Table 3. The results of this study confirmed [27] that water absorption increases with the addition of dietary fiber to cereal flour. Increased water absorption with higher bran ratio in the white flour could be explained by the hydroxyl group in fiber that allows water binding [16,28].

The dough development time of RWF was the shortest, and it increased as more bran was added. Fiber in the bran might prevent the gluten development of the dough [29,30]. However, no difference in dough stability was found among the samples. Dough characteristics such as water uptake, dough development time, and stability are known to be related to protein content and quality [31].

As shown in Table 3, the C2 and C3 values of the HMB significantly increased with the substitution ratio of bran, and the JMB was not significantly different from the RWF. The C2 is associated with protein weakening, and the C3 is associated with gelatinization, which indicates a viscosity peak. Reduced gliadin and glutenin contents in bran might promote gluten weakening [16]. However, no difference was found in C2 and C3 of JMB with the substitution ratio of bran. The C4 and C5 showed a tendency to decrease as the bran substitution ratio increased. The content of bran and water absorption increase proportionally, which delays the retrogradation of starch [32]. Banu et al. [16] reported that the index of retrogradation decreases as the content of wheat bran increases. In addition, Koksel et al. [32] reported that the specific volume of breads increases with the value of C4 and C5.

### 3.3. Characteristics of Bread

Data on the specific volume, crumb hardness, and color of bread made with various substitution ratios (0%, 5%, 10%, 15%, 20%, and 25%) of pulverized bran are outlined in Table 4. Depending on the replacement ratio of HMB or JMB, a significant difference was found in the specific volume of the bread. The specific volume of bread made with 5% and 10% HMB or JMB substituted flour was significantly higher than that of the RWF, which was used as a control in this study. On the other hand, the bread made with ≥15% HMB or JMB was low in specific volume, implying decreased baking quality when the bran was greater than 15%. Noort et al. [33] reported increased adverse effects on bread-making when the particle size of bran decreased. The results of this study confirm previous reports, but only when >15% of bran is used. The smaller particle size of wheat bran results in a higher surface area, thereby enhancing the fiber–protein interaction [33]. Hemdane et al. [6] reported that ferulic acid monomers bonding to the insoluble cell wall material may interfere with gluten network formation and have an adverse effect on baking. The results of this study showed that the volume of bread properly increased with ≤10% HMB or JMB substituted flour, even with the very small particle size bran, implying that volume is more affected by wheat variety than by particle size. In other studies, the addition of barley bran and rice bran increased the loaf volume of bread [34,35]. Among the lipids in the wheat bran, glycolipid binds with glutenin to help increase the bread volume [36,37]. Previous reports found that different effects of wheat bran depend on the type of wheat [38,39,40,41].

The hardness of the bread increased significantly with 25% HMB or 15%, 20%, and 25% JMB substitution (Table 4). Previous reports [32,42,43] showed that the higher amount and the smaller particle size of bran resulted in a harder crumb. The results of this study confirm that bread with JMB, which is small in particle size, is harder than bread with HMB when the substitution is ≥15%. As the substitution ratio increased, the L value significantly decreased, and a and b values tended to increase (see Table 4).

**Figure 2:**
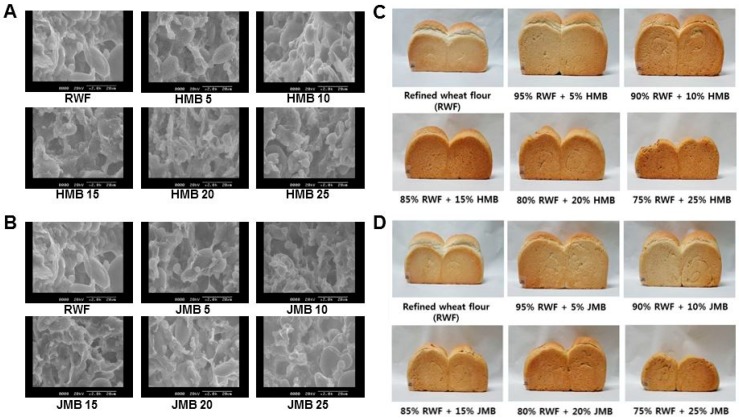
Scanning electron microscopy (SEM, ×2000) images of dough and bread made with various substitution ratios (0%, 5%, 10%, 15%, 20%, 25%) of (**A**,**C**) HMB or (**B**,**D**) JMB. HMB: bran pulverized by hammer mill, JMB: bran pulverized by jet mill.

The scanning electron microscopy (SEM) images of dough and bread made with various substitution ratios (0%, 5%, 10%, 15%, 20%, and 25%) of HMB or JMB are in Figure 2. The SEM image showed a more continuous gluten structure in dough with 5% and 10% HMB or JMB than in the control sample and dough with ≥20% HMB or JMB. This implies that <10% bran enhances baking property, which may be because of components such as phospholipids.

### 3.4. Antioxidant Activity of Bread

**Figure 3:**
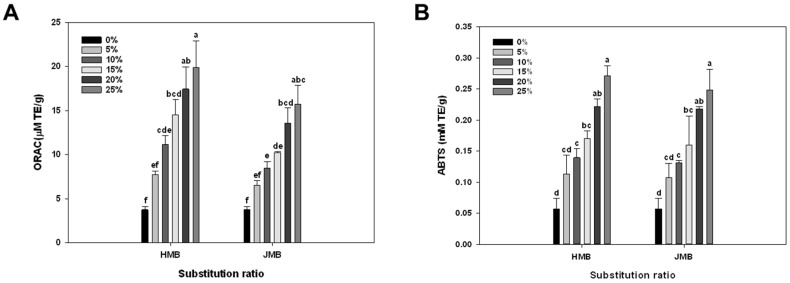
Effects of various substitution ratios of HMB or JMB on antioxidant activity of breads by (**A**) oxygen radical absorbance capacity (ORAC) and (**B**) 2, 2′-Azino-bis (3-ethylbenz-thiazoline-6-sulfonic acid (ABTS) assay. HMB: bran pulverized by hammer mill, JMB: bran pulverized by jet mill. Bars with different characters indicate a significant difference at *p* ≤ 0.05.

The antioxidant activity of bread with bran was evaluated by ORAC and ABTS and the results are in Figure 3. ORAC and ABTS are used to measure potential antioxidant capacity by measuring the defense from a free radical attack [44]. As shown in Figure 3, the antioxidant activity was significantly increased with the substitution ratio of bran. In both HMB and JMB, the antioxidant activity was the highest at 25% of bran substitution ratio. Many enzymes in wheat are involved in antioxidant activity, and most of these enzymes are distributed in bran [45]. Gunenc et al. [46] reported antioxidant activity in bread with 30% wheat bran and 2% alkylresorcinol extract.

Antioxidants in cereals are mostly phenolic compounds in the form of phenolic acids and insoluble bound ferulic acid, and they are concentrated in wheat bran [47]. The ferulic acid in particular is typically present in a variety of plants, particularly wheat in the form that is combined with the cell wall. Ferulic acid is known to have an antioxidant effect as a mechanism to remove free radicals by donating electrons or attacking free radicals in structure [48]. The addition of rice bran at 5%–10% improved the physical and antioxidant activity of rice bread [49]. Taken together, the antioxidant activity of bread increased with the addition of cereal bran.

## 4. Conclusions

The effects of the substitution ratio (0%–25%) and milling type of bran on the characteristics of dough and bread were investigated. In this study, flour substituted with <10% bran enhanced the bread regardless of the milling method quality by increasing specific volume and decreasing the hardness of bread. The addition of wheat bran with a large particle size milled with a hammer mill improved bread quality without affecting color or hardness, which could affect preference more effectively than the jet mill. HMB was more suitable for bread-making than JMB, implying a detrimental effect of the fine size of JMB. However, antioxidant activity increased by increasing the substitution ratio of bran regardless of the bran milling method. Overall, the results of this study imply that the proper addition of wheat bran enhances the physical and nutritional quality of bakery products.

## Figures and Tables

**Table 1:** The particle size and color characteristics of bran pulverized by hammer mill or jet mill.

Type of Bran	Mean Particle Size *** (µm)	Color		
		L ***	a ***	b ***
Bran	>150	64.07 ± 0.31 ^c^	6.90 ± 0.12 ^a^	18.36 ± 0.19 ^b^
HMB	119.71 ± 3.13 ^a^	74.42 ± 0.31 ^b^	4.64 ± 0.26 ^b^	19.86 ± 0.61 ^a^
JMB	25.78 ± 0.53 ^b^	78.37 ± 1.31 ^a^	2.51 ± 0.03 ^c^	15.42 ± 0.18 ^c^

JMB: bran pulverized by jet mill, HMB: bran pulverized by hammer mill. All values are means of three replications ± standard deviation. *** Indicates a significant difference at *p* ≤ 0.001 and different characters (a, b, c) in the same column indicate a significant difference at *p* ≤ 0.05.

**Table 2:** Protein, TDF, wet gluten, ash, and color characteristics of flours substituted with HMB or JMB at various levels (0%, 5%, 10%, 15%, 20%, and 25%).

Type of Bran	Bran (%)	Protein (%, db) ***	TDF (%, db) ***	Wet Gluten (%, 14% mb) ***	Ash (%, db) ***	Color		
						L ***	a ***	b ***
RWF	0	10.75 ± 0.04 ^h^	1.80 ± 0.19 ^j^	24.70 ± 0.15 ^a^	0.67 ± 0.10 ^h^	94.24 ± 0.35 ^a^	0.27 ± 0.03 ^g^	8.86 ± 0.05 ^e^
HMB	5	11.86 ± 0.10 ^g^	4.01 ± 0.34 ^i^	23.05 ± 1.12 ^ab^	0.74 ± 0.11 ^gh^	91.97 ± 0.56 ^b^	0.89 ± 0.10 ^f^	8.93 ± 0.33 ^e^
	10	12.25 ± 0.06 ^e^	7.19 ± 0.37 ^g^	23.14 ± 1.88 ^ab^	1.10 ± 0.07 ^f^	91.55 ± 0.44 ^bc^	1.23 ± 0.24 ^de^	9.97 ± 0.22 ^d^
	15	12.56 ± 0.06 ^c^	9.54 ± 0.36 ^e^	19.80 ± 0.41 ^cde^	1.36 ± 0.09 ^de^	90.33 ± 0.25 ^cd^	1.68 ± 0.24 ^bc^	11.39 ± 0.32 ^c^
	20	12.86 ± 0.02 ^b^	14.21 ± 0.61 ^b^	18.26 ± 0.52 ^de^	1.61 ± 0.04 ^bc^	88.57 ± 1.19 ^ef^	1.98 ± 0.36 ^a^	11.08 ± 0.46 ^c^
	25	13.17 ± 0.12 ^a^	15.61 ± 0.22 ^a^	18.79 ± 0.27 ^de^	1.88 ± 0.13 ^a^	88.44 ± 0.55 ^ef^	1.79 ± 0.06 ^ab^	10.96 ± 0.44 ^c^
JMB	5	12.08 ± 0.04 ^f^	2.42 ± 0.47 ^j^	21.91 ± 0.82 ^abc^	0.73 ± 0.08 ^gh^	89.60 ± 0.16 ^d^	0.88 ± 0.06 ^f^	10.35 ± 0.21 ^d^
	10	12.10 ± 0.05 ^e^	6.07 ± 0.38 ^h^	20.55 ± 0.96 ^bcde^	0.96 ± 0.10 ^fg^	89.11 ± 0.39 ^de^	1.16 ± 0.04 ^e^	11.34 ± 0.03 ^c^
	15	12.36 ± 0.05 ^de^	8.23 ± 0.25 ^f^	20.64 ± 1.74 ^bcd^	1.16 ± 0.05 ^ef^	88.64 ± 0.45 ^ef^	1.42 ± 0.06 ^cde^	12.16 ± 0.21 ^b^
	20	12.51 ± 0.05 ^cd^	10.69 ± 0.32 ^d^	17.67 ± 0.86 ^e^	1.47 ± 0.03 ^cd^	88.24 ± 0.18 ^f^	1.47 ± 0.01 ^cd^	12.22 ± 0.07 ^b^
	25	12.79 ± 0.03 ^b^	13.12 ± 0.13 ^c^	13.63 ± 0.67 ^f^	1.84 ± 0.02 ^ab^	87.84 ± 0.61 ^f^	1.65 ± 0.04 ^bc^	12.87 ± 0.10 ^a^

JMB: bran pulverized by jet mill, RWF: refined wheat flour, HMB: bran pulverized by hammer mill, TDF: total dietary fiber content. All values are means of three replications ± standard deviation. Protein, TDF, and ash were calculated based on dry weight (db). Wet gluten was calculated based on 14% moisture content (mb). *** Significantly differ at *p* ≤ 0.001. Different characters (a–j) in the same column indicate a significant difference at *p* ≤ 0.05.

**Table 3:** Water absorption, stability, and dough development by Mixolab^®^ analysis of flours substituted with HMB or JMB at various levels (0%, 5%, 10%, 15%, 20%, and 25%).

Type of Bran	Bran (%)	Torque (Nm)					Water Absorption (%) ***	Stability (min)	Dough Development Time (min) **
		C1 **	C2 ***	C3 *	C4 ***	C5 **			
RWF	0	1.12 ^bcde^	0.47 ^d^	1.82 ^d^	1.32 ^c^	2.69 ^de^	58.10 ± 0.42 ^e^	7.19 ± 0.97	1.70 ± 0.49 ^d^
HMB	5	1.08 ^e^	0.50 ^cd^	1.86 ^bcd^	1.75 ^ab^	3.06 ^ab^	59.00 ± 0.28 ^e^	8.18 ± 0.21	2.73 ± 0.94 ^bcd^
	10	1.12 ^bcde^	0.54 ^bc^	1.91 ^abc^	1.76 ^a^	2.95 ^abc^	61.05 ± 0.64 ^d^	8.18 ± 0.18	3.50 ± 0.18 ^bcd^
	15	1.13 ^bcd^	0.54 ^bc^	1.91 ^abc^	1.68 ^ab^	2.71 ^cde^	62.55 ± 0.78 ^c^	8.24 ± 0.08	3.51 ± 0.20 ^bcd^
	20	1.15 ^abc^	0.57 ^ab^	1.93 ^ab^	1.67 ^ab^	2.72 ^cde^	64.15 ± 0.49 ^b^	8.19 ± 0.09	5.06 ± 0.18 ^ab^
	25	1.20 ^a^	0.60 ^a^	1.96 ^a^	1.64 ^b^	2.73 ^cde^	65.40 ± 0.42 ^b^	7.43 ± 0.42	6.33 ± 0.22 ^a^
JMB	5	1.10 ^de^	0.50 ^cd^	1.87 ^bcd^	1.78 ^a^	3.09 ^a^	59.15 ± 0.35 ^e^	7.95 ± 0.14	2.11 ± 0.33 ^cd^
	10	1.10 ^cde^	0.47 ^d^	1.84 ^cd^	1.75 ^ab^	3.03 ^ab^	60.85 ± 0.35 ^d^	7.82 ± 0.69	3.54 ± 0.23 ^bcd^
	15	1.16 ^ab^	0.48 ^d^	1.85 ^cd^	1.76 ^a^	2.93 ^abcd^	64.25 ± 1.06 ^b^	7.14 ± 0.40	4.23 ± 0.32 ^abc^
	20	1.12 ^bcde^	0.46 ^d^	1.83 ^cd^	1.73 ^ab^	2.83 ^bcde^	65.10 ± 0.14 ^b^	7.05 ± 0.53	4.14 ± 0.44 ^abcd^
	25	1.15 ^abc^	0.47 ^d^	1.82 ^d^	1.69 ^ab^	2.65 ^e^	67.60 ± 0.85 ^a^	7.48 ± 0.88	4.56 ± 0.39 ^abc^

JMB: bran pulverized by jet mill, RWF: refined wheat flour, HMB: bran pulverized by hammer mill. C1: an objective value of 1.1 Nm on average, C2: the lowest viscosity of the dough after the start of heating, C3: the starch gelatinization and represents the maximum torque after C2, C4: stability, C5: the torque value that appears after cooling the dough and observing starch degradation. All values are means of two replications ± standard deviation. *, **, *** significantly differ at *p* ≤ 0.01, 0.05, 0.001, respectively. Different characters (a–e) in the same column indicate a significant difference at *p* ≤ 0.05.

**Table 4:** Specific volume (SV), crumb hardness, and color characteristics of breads made with flour substituted with HMB or JMB at various levels (0%, 5%, 10%, 15%, 20%, and 25%).

Type of Bran	Bran (%)	Bread		Color of Crumb		
		SV (mL/g) ***	Hardness (N) ***	L ***	a ***	b ***
RWF	0	3.12 ± 0.07 ^c^	7.22 ± 0.23 ^de^	80.93 ± 0.80 ^a^	−0.64 ± 0.11 ^g^	14.49 ± 0.14 ^f^
HMB	5	3.53 ± 0.05 ^a^	4.36 ± 0.03 ^e^	74.48 ± 1.25 ^ab^	1.44 ± 0.36 ^ef^	18.22 ± 0.73 ^e^
	10	3.42 ± 0.06 ^b^	4.81 ± 0.08 ^e^	73.89 ± 0.40 ^b^	2.13 ± 0.21 ^e^	18.94 ± 0.77 ^e^
	15	3.01 ± 0.02 ^d^	8.17 ± 0.14 ^d^	72.79 ± 0.51 ^bc^	2.97 ± 0.36 ^d^	22.15 ± 0.97 ^cd^
	20	2.98 ± 0.02 ^d^	8.23 ± 0.33 ^d^	69.62 ± 0.75 ^bc^	4.46 ± 0.17 ^b^	24.63 ± 0.47 ^ab^
	25	2.62 ± 0.02 ^f^	21.49 ± 0.56 ^b^	65.92 ± 0.66 ^c^	5.65 ± 0.19 ^a^	25.31 ± 0.20 ^a^
JMB	5	3.49 ± 0.01 ^ab^	5.90 ± 0.25 ^de^	74.75 ± 0.14 ^ab^	1.15 ± 0.24 ^f^	18.85 ± 0.76 ^e^
	10	3.44 ± 0.02 ^ab^	6.03 ± 0.20 ^de^	71.19 ± 6.81 ^bc^	2.12 ± 0.19 ^e^	21.47 ± 0.28 ^d^
	15	2.67 ± 0.01 ^ef^	15.12 ± 0.30 ^c^	71.09 ± 2.27 ^bc^	3.67 ± 0.29 ^cd^	23.32 ± 0.72 ^bc^
	20	2.77 ± 0.02 ^e^	14.88 ± 3.52 ^c^	70.11 ± 0.55 ^bc^	4.32 ± 0.20 ^bc^	24.77 ± 0.09 ^ab^
	25	2.02 ± 0.01 ^g^	26.10± 0.49 ^a^	66.50 ± 0.63 ^c^	5.85 ± 0.24 ^a^	25.60 ± 0.44 ^a^

JMB: bran pulverized by jet mill, RWF: refined wheat flour, HMB: bran pulverized by hammer mill. All values are means of three replications ± standard deviation. *** Significantly differ at *p* ≤ 0.001. Different characters (a–g) in the same column indicate a significant difference at *p* ≤ 0.05.
